# Pelvis-Toe Distance: 3-Dimensional Gait Characteristics of Functional Limb Shortening in Hemiparetic Stroke

**DOI:** 10.3390/s21165417

**Published:** 2021-08-11

**Authors:** Koshiro Haruyama, Michiyuki Kawakami, Kohsuke Okada, Kohei Okuyama, Keita Tsuzuki, Meigen Liu

**Affiliations:** Department of Rehabilitation Medicine, School of Medicine, Keio University, Tokyo 160-8582, Japan; k.haruyama.us@juntendo.ac.jp (K.H.); k.okada.pt@gmail.com (K.O.); okuyamak.pt@gmail.com (K.O.); keita.t5391@gmail.com (K.T.); meigenliu@keio.jp (M.L.)

**Keywords:** hemiplegia, gait analysis, stroke rehabilitation, lower extremity, gait disorders, neurologic

## Abstract

We aimed to investigate whether a newly defined distance in the lower limb can capture the characteristics of hemiplegic gait compared to healthy controls. Three-dimensional gait analyses were performed on 42 patients with chronic stroke and 10 age-matched controls. Pelvis-toe distance (PTD) was calculated as the absolute distance between an anterior superior iliac spine marker and a toe marker during gait normalized by PTD in the bipedal stance. The shortening peak during the swing phase was then quantified as PTDmin. The sagittal clearance angle, the frontal compensatory angle, gait speed, and the observational gait scale were also collected. PTDmin in the stroke group showed less shortening on the affected side and excessive shortening on the non-affected side compared to controls. PTDmin on the affected side correlated negatively with the sagittal clearance peak angle and positively with the frontal compensatory peak angle in the stroke group. PTDmin in stroke patients showed moderate to high correlations with gait speed and observational gait scale. PTDmin adequately reflected gait quality without being affected by apparent improvements due to frontal compensatory patterns. Our results showed that various impairments and compensations were included in the inability to shorten PTD, which can provide new perspectives on gait rehabilitation in stroke patients.

## 1. Introduction

Hemiplegic gait resulting from stroke often leads to a characteristic motion pattern and abnormal muscle activity, with a variety of abnormal joint trajectories [1]. Common features during the swing phase include decreased peak hip flexion, peak knee flexion-extension, and dorsiflexion [2,3]. These abnormal motion patterns primarily lead to a lack of foot-floor clearance and may also result in compensatory motion patterns [4,5]. Many patients with inadequate foot clearance produce compensatory motions such as circumduction, hip hiking, and vaulting [6], all of which increase the mechanical energetic cost [3].

Divergence between kinematic patterns and gait performance (including gait speed) has been shown in functional recovery after stroke [7,8]. Improvements in gait speed that might be achieved by compensatory patterns should not be equated with kinematic gait patterns as essential to recovery [4,9,10]. Kinematic analyses are recommended quantitative assessments to accurately identify gait patterns and multiple components [11,12]. However, general kinematic analysis deals with a large number of kinematic variables, depending on the degree of freedom of each joint [13,14]. In fact, single-joint and sagittal plane motions are of limited utility in explaining gait performance independently, and a global, multi-joint, multi-planar understanding is therefore required [15]. For example, in inverted pendulum motions during the stance phase [16] and self-impact double pendulum during the swing phase [17], which are known to be a mechanical property of gait, not only is the angle of the pendulum presumed to change characteristically during gait, but also the total length of the pendulum too. Quantifying the distance factor between coordinates to simply quantify joint trajectories in 3-dimensional space may facilitate our understanding of gait.

The concepts of functional limb shortening have been used as simple approaches to quantify joint trajectories during the swing phase of gait. Murray et al. [18] quantified the vertical distance of heel- and toe-floor clearance and reported that it was reduced in patients with drop foot and stroke patients. Moosabhoy et al. [19] quantified hip-toe distance as the sagittal-plane distance between the center of the hip and the toe, and Little et al. [20] reported a lack of that during the swing phase of hemiplegic gait. However, they did not reflect the 3-dimensional properties of gait and failed to isolate the various compensatory motion elements. In this study, we extended the effective length of the leg described in these previous studies to present values that reflect gait characteristics of the new inter-coordinate distances and examined their properties. We focused on the anterior superior iliac spine (ASIS) and toe coordinates for gait analyses to treat lower limb and pelvic motions comprehensively and defined pelvis-toe distance (PTD) as a linear distance in 3-dimensional space connecting these coordinates. We considered PTD as a clinically intuitive indicator providing a representative value of gait, separate from angular data.

Our hypothesis was that PTD during the swing phase would be shortest in mid-swing and is a factor unaffected by improvements due to compensatory motion patterns in the frontal plane. PTD might thus allow quantification of the quality of swing motion as integration of sagittal joint motion in the affected leg. The purpose of this study was to provide a kinematic representative value by quantifying PTD and to clarify the PTD characteristics of hemiplegic gait compared to that of healthy subjects.

## 2. Materials and Methods

### 2.1. Participants

Patients with hemiplegia following cerebrovascular accident and admitted to Keio University Hospital between May 2019 and March 2021 were enrolled in the study. To define a group of patients with a homogeneous level of motor functioning, inclusion criteria were: (1) diagnosis of stroke located in the cerebral hemisphere and resulting in sensorimotor disturbance on one side; (2) no evidence of hemianopsia; (3) no evidence of severe cognitive or language dysfunctions that would interfere with the ability to understand instructions; (4) no evidence of neglect; (5) at least 6 months after stroke; and (6) an ability to walk at least 10 m independently, without orthoses or assistive device. Non-disabled healthy controls were also recruited to serve as sex-, age-, height- and weight-comparable controls for stroke patients. Control participants exhibited normal leg joint range of motion and muscle strength, and did not show any apparent gait abnormalities. Demographic characteristics were recorded in all participants, and affected side and time after onset were collected in the stroke group.

This study was approved by the Medical Ethics Committee board of Keio University and conducted according to the principles expressed in the Declaration of Helsinki. All participants provided written informed consent prior to participation.

### 2.2. Procedure and Data Collection

An 8-camera motion capture system (Vicon Vantage V8; Vicon Motion Systems, Oxford, UK) was used to record 3-dimensional gait at 100 Hz. A standard Vicon Plug-in-Gait model was applied to the lower body with 16 reflective skin markers. The bipedal stance at rest was recorded for at least 3 s prior to the gait trials. Participants walked along a 10-m walkway at a comfortable, self-selected speed in at least 8 trials. Gait condition was barefoot without any assistive devices. Marker trajectories and joint angles were reconstructed, labeled, filtered, and modeled in Vicon Nexus version 2.8.2 motion capture software (Vicon Motion Systems).

All analyses were performed using an in-house code in MATLAB (version R2019a; The MathWorks, Natick, MA, USA). The mean of 10 stride data was normalized to the percentage gait cycle. Initial contact and toe off were estimated visually based on the y and z coordinates of the heel and toe markers, respectively. In addition, the timing of each identified gait event was rechecked on the animation of the lower limb model generated using another custom MATLAB script.

### 2.3. Kinematic Outcome Measures

The main outcome of this study was the PTD. A sagittal clearance (SC) angle and a frontal clearance (FC) angle were used as comprehensive lower limb angles for each plane to explain the PTD. The definitions and calculation methods of these variables are described below.

#### 2.3.1. PTD and PTDmin

**Figure 1:**
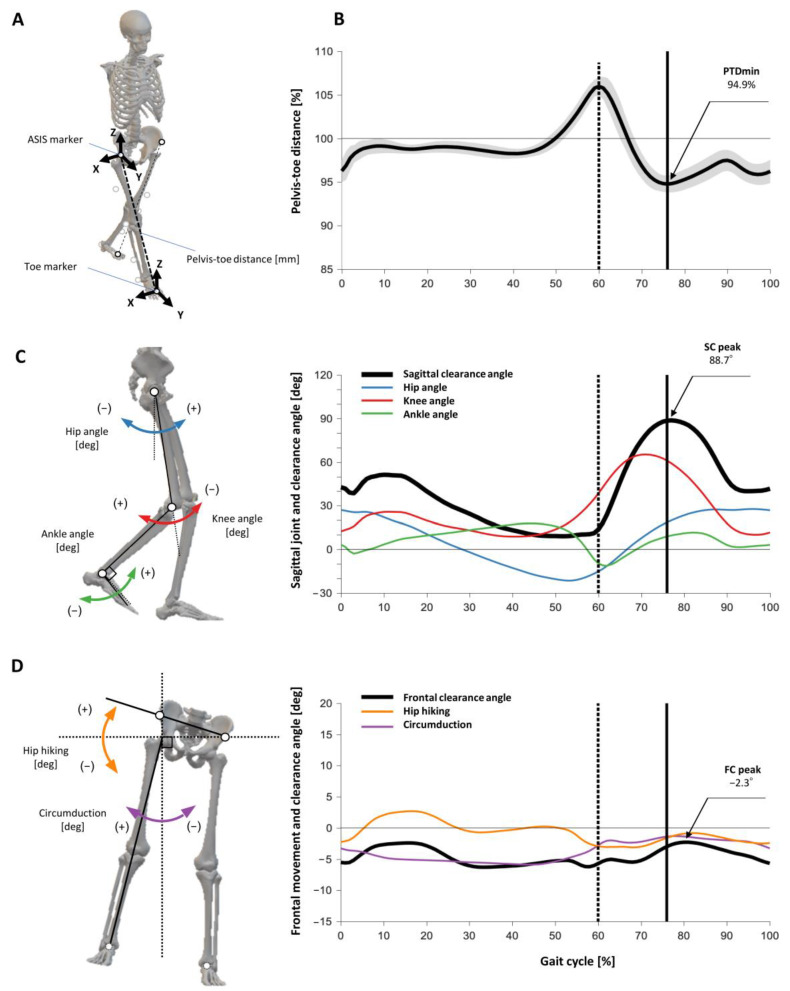
Indexes of gait outcomes in healthy controls. (**A**) Pelvis-toe distance (PTD) was the 3-dimensional distance between the unilateral ASIS and toe marker; (**B**) Plot of PTD during the gait cycle normalized by bilateral stance (%PTD). Mean plot was shown as black line, and standard deviations were shown in the gray range. PTDmin was defined as the first minimum peak value during the swing phase; (**C**) Sagittal angles of the lower limb and their definitions. Sagittal plane angle plots of the hip, knee, ankle joint, and summation of the sagittal clearance angle. The SC peak was defined as the maximum peak value during the swing phase. (**D**) Frontal angles of hip hiking and circumduction and their definition. Frontal plane angle plot of hip hiking, circumduction, and summation of the frontal clearance angle. The FC peak was defined as the maximum peak value during the swing phase. In (**B**–**D**), vertical dashed lines indicate toe-off timing and solid vertical lines indicate PTDmin timing.

PTD was calculated as a linear distance in 3-dimensional coordinate data between an ASIS marker and a toe marker (placed over the second metatarsal head) on each side (Figure 1A). PTD was derived from the following equation:

(1) $$\mathrm{PTD}=\sqrt{{\left(x_{ASIS}-x_{Toe}\right)}^{2}+{\left(y_{ASIS}-y_{Toe}\right)}^{2}+{\left(z_{ASIS}-z_{Toe}\right)}^{2}}.$$

The percentage PTD (%PTD) was calculated as the PTD during the gait cycle normalized to the PTD in bipedal stance. Finally, minimum %PTD (PTDmin) was defined as the first minimum peak value during the swing phase (Figure 1B). Although a second negative peak in the terminal swing was observed in some subjects, the first negative peak was adopted to provide the essence of foot clearance. Theoretically, in cases with PTDmin < 100%, foot clearance is maintained by functional limb shortening. Conversely, in cases with PTDmin > 100%, functional limb shortening is assumed to be insufficient and some compensatory motions are required to achieve foot clearance. PTDmin and its timing in the percentage gait cycle were also calculated and recorded.

#### 2.3.2. Index for the SC Angle

The SC angle was defined as the summation of sagittal plane angles of the three lower limb joints plotted across the gait cycle. The sagittal angle utilized a joint angle convention in which hip flexion, knee flexion, and ankle dorsiflexion were defined as “positive” joint rotations [19]. The ankle joint was set to 0 for the neutral ankle position. Since leg clearance increased with increased flexion angles, the maximum value during the swing phase was defined as the SC peak (Figure 1C). The SC peak and its timing in the percentage gait cycle were calculated as reflecting comprehensive motion in the sagittal plane. Maximum peak angles of the hip, knee, and ankle joints during the swing phase were also recorded.

#### 2.3.3. Index for the FC Angle

Hip hiking was defined as an excessive frontal plane pelvic angle and circumduction as an excessive frontal plane hip angle [6,15,21]. Absolute angles were defined as the vertical and horizontal reference axes in order to integrate hip hiking and circumduction in the same dimension. The index of hip hiking was the frontal angle between the line connecting the ASIS and horizontal line, and the representative value was the maximum peak during the swing phase [4]. The index of circumduction was the frontal angle between the line connecting the hip joint center and the heel marker as the vertical line, and the representative value was the maximum peak during the swing phase [6,21]. The FC angle was defined as the sum of two compensatory frontal plane angles plotted over the gait cycle, and the maximum value during the swing phase was defined as the FC peak (Figure 1D). The FC peak and its timing in the percentage gait cycle were calculated as representing comprehensive motion in the frontal plane. The maximum peak angles of the hip hiking and circumduction during the swing phase were also recorded.

### 2.4. Clinical Outcome Measures

Self-selected walking speed (SSWS) was measured in all participants. SSWS was calculated from walking time measured using a stopwatch on a 10-m straight walkway, with mean gait speed over three trials expressed in meters per second [22]. Stroke patients underwent the following clinical assessments: the Fugl-Meyer Assessment Lower Extremity Subscale (FMA-LE), the Modified Ashworth Scale (MAS), and the Gait Assessment and Intervention Tool (GAIT). The FMA-LE was primarily used to measure impairment of motor ability, consisting of a 34-point score [23,24]. The MAS comprises a 5-level scale to examine joint spasticity during passive muscle stretching [25]. The MAS was administered only for muscles of the lower limbs, specifically for the knee flexors and extensors, and the ankle plantar flexors. The GAIT is an observational gait assessment tool composed of 31 items divided into three sections: 4 items on the upper limb and trunk, 14 on the lower limb and trunk during the stance phase, and 13 on the swing phase [26]. The highest score possible for the scale is 62.

### 2.5. Statistical Analyses

Descriptive statistics were used to describe the demographic characteristics of participants. The Shapiro–Wilk test showed that data fitted a normal distribution and the Levene’s test showed that all homologous datasets were characterized by equal variance. Group differences in demographic characteristics were determined by Student’s *t*-test, except for sex, for which Fisher’s exact test was used. Kinematic data for right and left legs in the control group were combined after a paired *t*-test confirmed the absence of differences. Kinematic outcome measures were tested for differences using Student’s *t*-test for intergroup comparisons and the paired *t*-test for comparisons between the affected and non-affected sides of the stroke group. To determine the relationship of PTDmin to each pattern of plane joint motion, the association of PTDmin—the SC peak to PTDmin—the FC peak was calculated using Pearson’s correlation coefficient. Correlations between PTDmin on the affected side and SSWS and GAIT swing subscore were examined to clarify the relationship between PTDmin and gait parameters in the stroke group. Statistical analyses were conducted using SPSS-J version 23.0 (IBM Japan, Tokyo, Japan). The threshold for statistical significance was set at *p* = 0.05.

## 3. Results

Forty-two patients with chronic hemiparetic stroke and ten healthy controls were enrolled. Demographic characteristics and results of functional assessment and general gait parameters are summarized in Table 1. Demographic characteristics showed no difference between groups. SSWS was significantly slower in the stroke group than in age-matched controls (*p* < 0.01).

**Figure 2:**
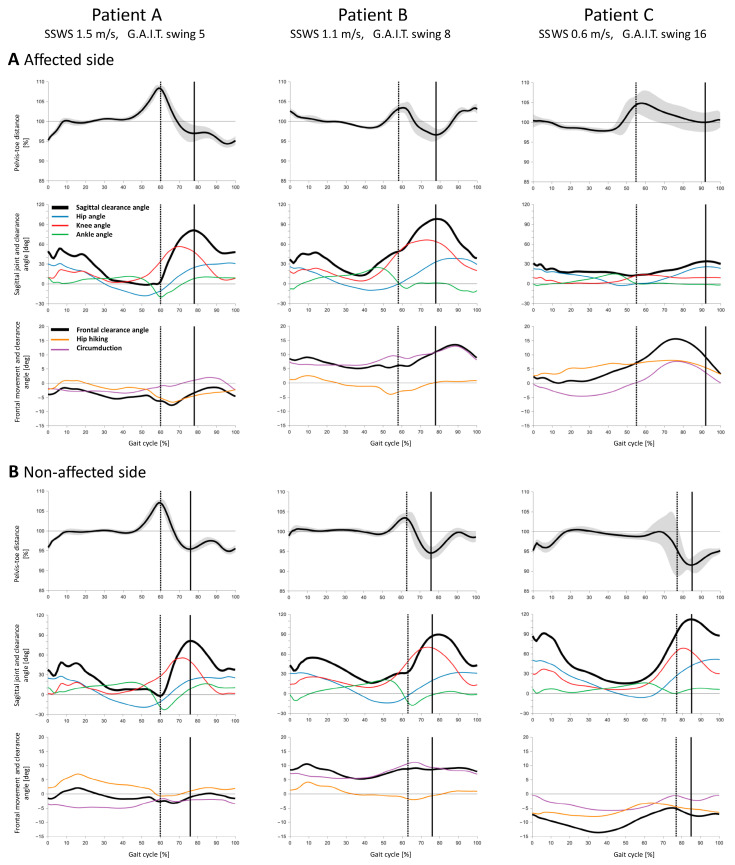
Kinematic parameters of affected side (**A**) and non-affected side (**B**) in stroke patients. PTD plots for representative cases of 3 stroke patients (**top**), plots for the sagittal plane joint angle and the SC angle (**middle**), and plots for the frontal compensatory motion angle and the FC angle (**bottom**) are shown. Abbreviations: SSWS, self-selected walking speed; GAIT; Gait Assessment and Intervention Tool.

Figure 2 shows representative findings from three stroke patients. The control group showed that all values were within normal range for sagittal joint angles with no frontal compensatory motion pattern, and the left and right PTD plot showed similar waveforms. Well-functioning cases in the stroke group displayed no frontal compensatory pattern, although PTDmin was slightly deficient on the affected side. In cases with low FMA-LE, the FC peak increased in a trade-off relationship with the decrease in the SC peak, compensating for the lack of PTDmin. The timing of PTDmin was almost identical to the SC peak.

Results for PTDmin, the FC peak, and the SC peak on the affected and non-affected sides in the stroke and control groups are summarized in Table 2. PTDmin was below 100% for both sides in the control group, with a value of 96.1% (standard deviation, 1.0). The stroke group showed less shortening on the affected side and excessive shortening on the non-affected side compared to controls. PTDmin above 100% was detected on the affected side in 23.8% (10/42) of stroke patients. For the stroke group, particularly on the affected side, PTDmin and its timing showed wide variance among patients. The FC peak was significantly excessive only on the affected side compared to other groups. Similar to increases in the FC peak, the respective peak angles of hip hiking and circumduction were also significantly greater on the affected side. FC peak timing occurred significantly earlier on the affected side compared to both the non-affected side and healthy controls. The SC peak was significantly lower on the affected side, and knee flexion was significantly lower. On the other hand, the SC peak, hip flexion, and ankle dorsiflexion occurred more excessively on the non-affected side than on the affected side and healthy controls.

**Figure 3:**
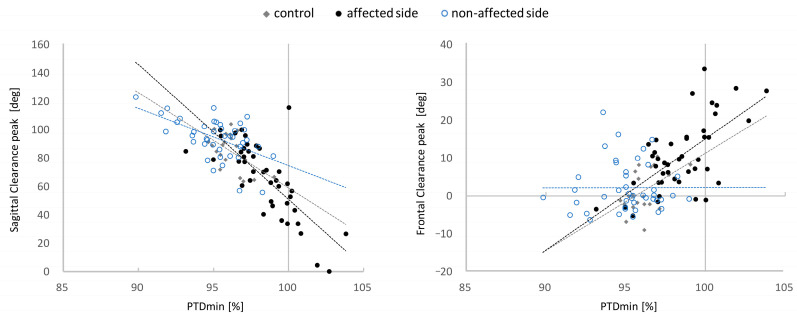
Correlations of PTDmin with the SC peak (**left**) and the FC peak (**right**). Dashed lines represent the trend line for each group (black is the affected side, blue is the non-affected side, and grey is the control).

**Figure 4:**
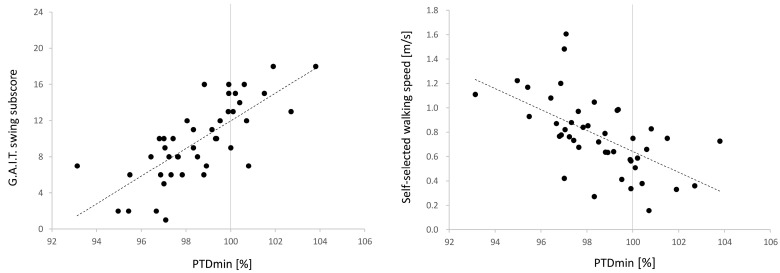
Correlation between PTDmin of affected side and the GAIT score during swing phase (**left**) and SSWS (**right**). The Dashed line represents the trend line on the affected side.

All groups showed negative correlations between PTDmin and the SC peak, as −0.77 (*p* < 0.01) in all participants, −0.55 (*p* = 0.01) in controls, −0.75 (*p* < 0.01) in the affected side, and −0.54 (*p* < 0.01) in the non-affected side (Figure 3A). PTDmin and the FC peak uniformly showed a positive correlation, with 0.57 (*p* < 0.01) in all participants, 0.48 (*p* = 0.03) in controls, and 0.65 (*p* < 0.01) in the affected side, with no correlation in the non-affected side of 0.01 (*p* = 0.99) (Figure 3B). PTDmin was also associated with gait outcomes. PTDmin correlated highly with the GAIT swing subscore (r = 0.75, 95% confidence interval (CI) 0.58–0.86, *p* < 0.01; Figure 4A). A correlation with the GAIT total score was also confirmed (r = 0.67, 95%CI 0.46–0.81, *p* < 0.001). Similarly, SSWS and PTDmin showed a moderate correlation (r = −0.59, 95%CI −0.75 to −0.36, *p* < 0.01; Figure 4B).

## 4. Discussion

This study investigated whether a newly defined distance between lower limb segments can capture the characteristics of a hemiplegic gait compared to age-matched controls. The novelty of the PTD is that it quantifies abnormal kinematic patterns into a simple and intuitive value using 3-dimensional distance. Absolute distance in 3-dimensional space can be argued to integrate more essential gait pattern information than variables that deal with only a single plane. Our results showed that PTDmin was associated with angular outcomes in the sagittal and frontal planes, as well as detecting differences between the affected and non-affected sides and healthy controls. PTDmin on the affected side correlated with gait quality and gait speed among stroke patients. PTDmin could manifest inadequate joint motions during the swing phase of the affected lower limb, since the affected and non-affected limbs also showed clear differences.

In addition, pelvic motion cannot be ignored for foot clearance [20,27]. The PTD uses the pelvis, rather than the hip joint, as the starting point for quantifying motions of the lower extremity. This allows the 3-dimensional motions of the pelvis and hip joint to be included in the PTD. Theoretically, PTD is shortened by pelvic anteversion, hip flexion, knee flexion, and ankle dorsiflexion. Conversely, these are extended by motions in the opposite direction. Swing leg clearance is commonly understood to be due to the simultaneous contributions of the hip, knee, and ankle joints [27]. Even backward pelvic rotation in the swing phase could compensate for hip flexion and decrease PTDmin [8]. In the normal gait pattern, PTD was maximal at push-off and minimal at mid-swing clearance. The present results confirmed a relationship between lower limb shortening and joint motion using the correlation coefficient between PTDmin and comprehensive joint motion. Since the range of motion of the affected leg was reduced in stroke patients [28], PTDmin was associated with reduced joint motions in the sagittal plane, consistent with our hypothesis. This promoted the understanding that sagittal leg joint angles that contribute to foot clearance directly contribute to leg shortening during the swing phase of the gait. Importantly, PTDmin responded to pure kinematic patterns in the sagittal plane without affecting the frontal compensatory patterns of the pelvis and lower extremity.

The normal PTDmin value was approximately 96% from age-matched controls and standard deviation was relatively uniform at 1%. This shows that normal toe clearance is achieved with a maximum reduction by about 4% compared to the bipedal stance. The values identified from both sides of stroke patients showed poor shortening or over-shortening of the effective length of the leg. In particular, PTDmin greater than 100% was specific to the affected side and was accompanied by compensatory strategies such as clearance contributions from frontal motions (Figure 2). This result reinforces previous studies that reported an association between a lack of sagittal joint motion and compensatory frontal plane motion [29]. PTDmin correlated more with the GAIT swing subscore than with gait speed. Gait speed is a typical outcome of walking ability, but does not necessarily reflect an optical kinematic pattern [5]. In fact, strategies to increase frontal compensatory patterns might be employed to improve gait speed [4,5]. The GAIT score is a semi-quantitative measure of the abnormal motion pattern based on the frontal and sagittal planes by observational gait analysis. PTDmin has been suggested as an index to quantify the quality of the kinematic pattern in the swing phase because of its high correlation with the GAIT swing subscore.

We found a significant difference in knee flexion and a representative SC peak between the control group and the affected side in the stroke group. The hip and ankle joints showed no difference, and knee flexion was considered as the main contributor to changes in lower limb length [20,29]. The exaggerated PTDmin, hip flexion, and ankle dorsiflexion on the non-affected side also appeared to represent compensatory bilateral abnormalities in stroke patients [20,28,29]. The lack of consistent trend may have been because the contributions of hip joint and ankle joint to clearance could trade off the limitations of each other [30]. The FC peak and the two frontal compensatory patterns showed clear differences between groups and were excessive on the affected side. An increase in frontal compensatory patterns did not lead to improvements in PTDmin, but a positive correlation with an increase in the FC peak was found in response to a lack of PTDmin. Therefore, PTDmin reflected both the lack of sagittal plane joint motion and the contribution of frontal plane compensatory motions on the affected side. Our results acknowledge the diversity of strategies used to achieve PTDmin among participants and suggest that inter-joint coordination of the swing leg might be consolidated in PTDmin.

In general, the gold standard for gait ability is gait speed [31,32]. However, gait speed has been pointed out to be insufficient to detect qualitative changes in stroke population [12,33]. Various outcomes of kinematics and kinetics to capture gait quality have been presented by researchers, as follows: muscle synergies [33], paretic propulsion [34,35], gait asymmetry [33,36], intralimb/interlimb coordination [37,38], gait deviation index [39,40], and gait variability index [39,41]. Nonetheless, no recommended outcomes have been determined at this time [12]. These measures are often considered to have limited clinical applicability due to the cumbersome procedures, time required, and limited plane and joint indices [42]. The setup for recording PTD only requires two marker coordinates per side, which may solve some of the clinical limitations to kinematic measures for minimal procedures using motion capture in clinical settings. The clinical significance of PTD lies in the fact that it provides a useful yet relatively simple parameter, the 3-dimensional measurement of lower limb length, and thus provides the possibility of capturing the characteristics of abnormal gait without the use of numerous markers.

Kinematic distance indices for the swing phase that have already been reported include maximal limb shortening [19,20], shortening of hip-toe length [29,43], leg length discrepancy [44], and minimal toe clearance [18,19,20,29,43]. All of these differ from the PTD, but do not seem to be in common use. The characteristics of these distance factors were based on the assumption that coordinated motions of multiple lower limb joints change the effective length of the lower limb [19]. In fact, intralimb coordination of lower limb segments has been shown to be characteristically impaired in stroke even after eliminating the effect of gait speed [45]. Maximal limb shortening and shortening of hip-toe length were calculated based on sagittal plane distance between the hip joint and toe. The former represented shortening of the distance between the hip joint center and the second metatarsal head during stance, calculated by instantaneous hip-toe distance divided by instantaneous distance from the hip joint center to the floor. The latter used a simple set of markers to define the vertical distance between the hip joint marker and fifth metatarsal head marker, and was calculated as changes in limited values between mid-stance and mid-swing at the timing of ankle crossing in the stance and swing phases, respectively. The contributions of the pelvis and hip joints to these indices remain unknown, and deviated motion on the frontal plane was considered a limited index that cannot be directly captured from different planes. These issues had also been raised by the authors regarding the meaning of 3-dimensional extension [19]. Leg length discrepancy represents a discrepancy in lower limb length and has mainly been used in the context of orthopedic and developmental problems. Khamis et al. [44] took the absolute distance between hip and ankle joint centers, the heel, and the second metatarsal head, and confirmed the effect of structural lower limb length during gait in healthy subjects. This study differed from our study in that it did not include the effect of the pelvis, used a different method of normalization, and had a different purpose. Nevertheless, the findings were similar to those of our study, in that the absolute distance between the hip joint center and second metatarsal head changed dynamically during gait, and the absolute distance of the lower limb in 3-dimensional space provided a new perspective to capture the functional kinematics of gait. Minimal toe clearance was an essential and clinical indicator because it measured the absolute distance from the floor during stance, but did not represent the strategy of clearance [18]. Furthermore, this value has been noted to be difficult to detect in stroke patients [20,29,43]. In contrast, PTDmin was detectable in all participants and could be compared with normal gait. However, it is important to note that PTDmin is not an indicator of foot clearance. Matsuda [29] and Burpee [46] have shown that foot clearance is directly related to leg shortening in stroke patients. We believe that PTDmin is applicable when the goal is to capture pathological kinematic features of the swing phase.

A key strength of PTDmin is that it eliminates frontal compensatory patterns that cannot be captured from sagittal plane measurements alone. In other words, because frontal compensation on the affected side did not contribute, PTDmin was able to distinguish and quantify strategies to achieve swing by hip hiking and circumduction. The Stroke Recovery and Rehabilitation Roundtable proposed the importance of distinguishing between restitution and compensation by requiring kinematic and kinetic measures as indexes of motor recovery [12,47]. This means that PTDmin could be used as an outcome to capture the restitution of swing pattern in stroke patients. For instance, applying PTD to gait analyses with and without an ankle orthosis may help quantify the effects of the orthosis on foot clearance [43]. To facilitate use in clinical practice, the possibility of substituting PTD measurements using depth sensors [48] and wearable sensors [49] must be verified in the future.

Several limitations to this study must be kept in mind when interpreting our findings. First, PTDmin might not reflect all compensatory motions. For example, gait abnormalities, such as vaulting, foot inversion, or ipsilateral pelvic drop of the contralateral leg (Trendelenburg sign), may not contribute much to the shortening of the PTD. In addition, since FC and SC angles were all customed outcomes, their validity needs to be verified. Finally, consideration should be given to generalization, since the population of this result was based on a limited sample obtained using selection criteria.

## 5. Conclusions

In this study, lower limb shortening index during the swing phase using the absolute distance between the pelvis and toe revealed gait characteristics of both the chronic stroke patients and healthy controls. PTDmin, the percentage of limb shortening normalized to the bipedal stance, was high (>100%) on the affected side in some patients in the stroke group, but it was low (always <100%) in the control group and on the non-affected side in patients in the stroke group. Furthermore, PTDmin seems to adequately reflect the quality of gait by correlating with the stroke-specific abnormal gait scale, without being affected by the apparent improvement due to frontal compensatory patterns. Our results show that various impairments and compensations are included in the inability to shorten the PTD by only 4%, which can provide a new perspective for gait rehabilitation in stroke patients.

## Figures and Tables

**Table 1:** Demographic characteristics and clinical measures.

	Stroke	Control	*p* Value
Number of subjects	42	10	
Age [years]	52.3 (13.0)	51.9 (14.8)	0.93
Sex [F/M]	12/32	5/5	0.26
Height [cm]	166.5 (6.9)	165.8 (7.1)	0.77
Weight [kg]	62.8 (9.6)	64.3 (14.1)	0.68
Affected side [R/L]	21/23	-	
Time after onset [months]	42.5 (33.6)	-	
FMA-LE [0–34]	25.9 (3.8)	-	
MAS quadriceps [0–5]	0 (0, 1)	-	
MAS hamstrings [0–5]	0 (0, 1)	-	
MAS gastrocnemius [0–5]	1 (0.25, 2)	-	
SSWS [m/s]	0.8 (0.3)	1.4 (0.1)	<0.01
GAIT total score [0–62]	23.3 (8.9)	-	
GAIT swing subscore [0–24]	9.8 (4.4)	-	

Reported values represent mean (standard deviation) or median (25th, 75th percentiles). Abbreviations: FMA-LE, Fugl-Meyer Assessment Lower Extremity Subscale; MAS, modified Ashworth scale; SSWS, self-selected walking speed; GAIT.; Gait Assessment and Intervention Tool.

**Table 2:** Summary of kinematic results.

		Stroke							Control	
		Affected Side			Non-Affected Side				Both Sides	
PTDmin	[%]	98.5	(2.1)	**	95.0	(2.0)	*	‡	96.1	(1.0)
PTDmin timing	[%gait cycle]	79.6	(9.2)		81.5	(4.8)	**		76.5	(1.8)
Frontal clearance peak	[deg]	10.6	(9.5)	**	2.2	(6.5)		‡	1.4	(5.8)
Frontal clearance peak timing	[%gait cycle]	82.3	(6.1)	**	88.2	(10.6)		‡	88.5	(8.5)
Hip hiking angle peak	[deg]	4.5	(4.7)	**	0.1	(2.5)		‡	0.6	(1.2)
Circumduction angle peak	[deg]	6.7	(6.5)	**	2.8	(6.5)		‡	1.7	(6.3)
Sagittal clearance peak	[deg]	65.9	(26.3)	**	94.2	(14.1)	*	‡	84.9	(12.1)
Sagittal clearance peak timing	[%gait cycle]	78.2	(6.1)		82.6	(4.3)	**	‡	78.6	(1.7)
Hip flexion angle peak	[deg]	28.3	(8.1)		34.9	(6.8)	*	‡	30.2	(6.6)
Knee flexion angle peak	[deg]	42.0	(5.8)	**	65.2	(9.5)		‡	65.2	(5.3)
Ankle dorsiflexion angle peak	[deg]	5.8	(7.8)		9.5	(5.2)	*	‡	6.2	(4.7)

Reported values represent mean (standard deviation) * <0.05, ** <0.01 compared to controls; ‡ <0.01 between affected and non-affected sides. Abbreviations: PTDmin, minimum percentage pelvis-toe distance.

## Data Availability

The datasets generated during and/or analyzed during the current study are not publicly available for secondary use due to build the single center-specific gait database, but are available from the corresponding author on reasonable request.

## Abbreviations

ASIS	anterior superior iliac spine
CI	confidence interval
FC	frontal clearance
FMA-LE	Fugl-Meyer Assessment Lower Extremity Subscale
GAIT	Gait Assessment and Intervention Tool
MAS	modified Ashworth scale
PTD	pelvis-toe distance
PTDmin	minimum %pelvis-toe distance
SC	sagittal clearance
SSWS	self-selected walking speed
